# Reviewers BJCVS 32.6

**Published:** 2017

The Brazilian Journal of Cardiovascular Surgery (BJCVS) is grateful for the reviewers, listed below, which collaborated in this edition. Without their work would be impossible to keep the high scientific standard of our journal.

Alexandre Hueb
Ana Maria Silva
Anthony Panos
Celso Salgado de Melo
Chiara Gatto
Edmo Atique Gabriel
Eduardo Keller Saadi
Fabio Gaiotto
Fábio Taniguchi
Fernando Antoniali
Gabriel Liguori
Gilberto Venossi Barbosa
João de Deus e Brito
José Carlos Pachón
Lindemberg Silveira
Luís Alberto Oliveira Dallan
Luiz Antônio Castilho Teno
Luiz Antônio Rivetti
Marcos Aurélio Barboza de Oliveira
Maurício Machado
Melchior Lima
Michel Pompeu Barros de Oliveira Sá
Nicolas Brozzi
Paulo Roberto B. Évora
Reinaldo Bestetti
Ricardo Dias
Ricardo de Carvalho Lima
Tania Strabelli

